# SFARI genes and where to find them; modelling Autism Spectrum Disorder specific gene expression dysregulation with RNA-seq data

**DOI:** 10.1038/s41598-022-14077-1

**Published:** 2022-06-16

**Authors:** Magdalena Navarro Torres Arpi, T. Ian Simpson

**Affiliations:** 1 School of Informatics, University of Edinburgh, 10 Crichton Street, Edinburgh, EH8 9AB UK; 2 Simons Initiative for the Developing Brain (SIDB), Centre for Brain Discovery Sciences, University of Edinburgh, Edinburgh, UK

**Keywords:** Computational biology and bioinformatics, Neuroscience, Diseases

## Abstract

Autism Spectrum Disorders (ASD) have a strong, yet heterogeneous, genetic component. Among the various methods that are being developed to help reveal the underlying molecular aetiology of the disease one approach that is gaining popularity is the combination of gene expression and clinical genetic data, often using the SFARI-gene database, which comprises lists of curated genes considered to have causative roles in ASD when mutated in patients. We build a gene co-expression network to study the relationship between ASD-specific transcriptomic data and SFARI genes and then analyse it at different levels of granularity. No significant evidence is found of association between SFARI genes and differential gene expression patterns when comparing ASD samples to a control group, nor statistical enrichment of SFARI genes in gene co-expression network modules that have a strong correlation with ASD diagnosis. However, classification models that incorporate topological information from the whole ASD-specific gene co-expression network can predict novel SFARI candidate genes that share features of existing SFARI genes and have support for roles in ASD in the literature. A statistically significant association is also found between the absolute level of gene expression and SFARI’s genes and Scores, which can confound the analysis if uncorrected. We propose a novel approach to correct for this that is general enough to be applied to other problems affected by continuous sources of bias. It was found that only co-expression network analyses that integrate information from the whole network are able to reveal signatures linked to ASD diagnosis and novel candidate genes for the study of ASD, which individual gene or module analyses fail to do. It was also found that the influence of SFARI genes permeates not only other ASD scoring systems, but also lists of genes believed to be involved in other neurodevelopmental disorders.

## Introduction

Autism Spectrum Disorder (ASD) encompasses a diverse group of developmental disorders characterised by deficits in social interaction, impaired communication skills, and a range of stereotyped and repetitive behaviours^1^. ASD has a strong genetic component, with heritability estimated to be as high as 52%^2^ and hundreds of genes believed to be disrupted by it^3^, however, for 75% of the cases, the causes still remain unknown^4^, which suggests there is still a lot to discover about this complex and heterogeneous disorder.

There are many approaches to study the genetic components underlying the aetiology of ASD. The most direct, and one of the most popular approaches, is to study likely causative mutations that have been found in patients with the disorder. Arguably the largest source of these are the Simons Foundation Autism Research Initiative (SFARI)^5^ who created SFARI-gene, a constantly evolving, expertly curated database of candidate genes involved in autism susceptibility by integrating genetic information from multiple research studies. The latest version of the dataset consists of 942 genes, which have been scored with a value from 1 to 3 reflecting the strength of the evidence linking a gene to ASD, where a score of 1 is assigned to genes that have a high confidence of being implicated, 2 to strong candidates, and 3 to genes that only have relatively weak evidence supporting their connection to ASD.

Another common approach is to compare gene expression between ASD patients and unaffected controls using transcriptomics. This has led to the discovery of many candidate genes for ASD and identified convergent molecular processes involved in the disorder^6^. These analyses have also revealed interactions between molecular pathways and other contributory factors and have helped us to understand how diverse mechanisms and risk factors can combine to produce complex behavioural outcomes^4^.

Interpreting transcriptomic data in the context of the curated SFARI-gene list is commonly undertaken both in experimental design to validate results^7–14^, and, more recently, to combine information from these two sources into single models that learn jointly from these data using classification methods or network analysis tools^15–18^.

These classification methods use transcriptomic datasets derived from neurotypical donors and therefore model canonical gene expression patterns in the brain. We believe that using a combined analysis of transcriptomic data derived from ASD donors and unaffected controls instead, can provide new insights into ASD, including revealing patterns of ASD-specific dysregulation and potentially novel ASD candidate genes. Our aim is to determine how best to combine transcriptomic data from both ASD and unaffected patients alongside SFARI genes; focusing on when it is suitable to combine them and what aspects should be taken into consideration when doing so.

In this study we analyse an ASD-specific gene co-expression network at three different levels of granularity starting at the *gene-level*, by examining individual genes independently from one another, then at the *module-level*, by examining groups of genes defined by similarities in their expression profiles, and finally at the *systems-level*, by analysing all of the genes simultaneously in a fully-connected co-expression network.

## Results

### SFARI genes have higher levels of expression than other neuronal and non-neuronal genes

**Figure 1:**
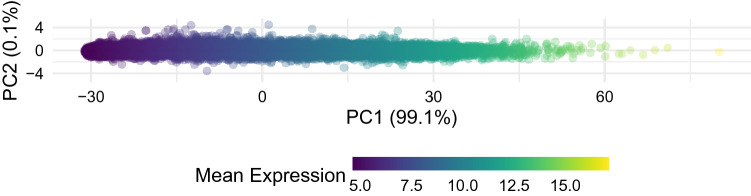
Mean level of expression plays a central role in gene characterisation. PCA plot of genes characterised by their expression patterns across all samples and coloured by their mean level of expression. The numbers in parenthesis on the axis represent the percentage of variance explained by each component. The x-axis corresponds to the first principal component, which represents over 99% of the information in the dataset and is strongly related to the mean level of expression of the genes.

Before studying more specific patterns in gene expression related to SFARI genes and ASD we perform a principal component analysis of gene expression across all 80 samples and find that 99% of variation is captured in the first principal component. We find a perfect correlation between this first principal component and the mean level of gene expression. This can be clearly seen in Fig. 1 where genes are coloured by their mean level of expression. This means that the ASD diagnostic status of a sample is not a dominant feature at this level of analysis and that more sensitive approaches will be needed to investigate the relationship between gene expression and ASD.

**Figure 2:**
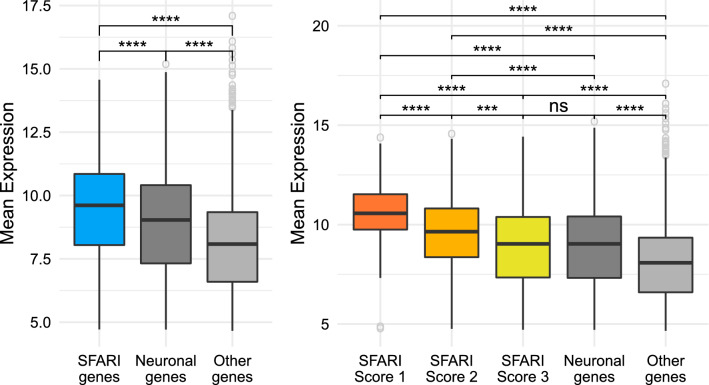
SFARI genes have higher levels of expression than other genes. Comparison between the SFARI genes, genes with neuronal annotations and with the rest of the genes in the dataset. The brackets at the top indicate pairwise comparisons, using a Welch t-test to study wether the differences in level of expression between groups are statistically significant, and the asterisks indicate the magnitude of the corrected p value of each test: ns = p value $\ge$ 0.05, *p value < 0.5, **p value < 0.01, ***p value < 0.001, and ****p value < 0.0001. (**A**) SFARI genes. (**B**) SFARI Scores. Outlier genes are represented individually as open circles. The t-tests use all the points in each group, including outliers.

Comparing the mean level of expression of the genes that correspond to SFARI against the rest of the genes in our transcriptomic dataset, we can see that they have a statistically significantly higher level of expression than both of the other gene groups with a Benjamini–Hochberg corrected p value lower than $10^{-4}$, as seen in Fig. 2A, agreeing with the results presented in^18^.

Figure 2B shows that separating the SFARI Genes by SFARI Scores, we find a similar pattern; the higher the SFARI Score, the higher the level of expression of the genes, with genes belonging to SFARI Score 1 having the highest level of expression of all groups, followed by SFARI Score 2 and then by SFARI Score 3. All of the differences between groups are statistically significant with a corrected p value lower than $10^{-3}$, even between SFARI Scores, except for the comparison between SFARI Score 3 and the neuronal genes, where the difference is not statistically significant.

There is as yet no biological or technical explanation for the observed relationship between a SFARI gene’s mean level of expression and its role in ASD. We have modelled the effect of a range of possible features of the samples as co-variates, but none of these explain the effect. Taken together, the previous report of elevated gene expression in SFARI genes^18^ and our finding of the same pattern in three independent ASD gene expression datasets supports the idea that there is a group of neuronal genes associated with ASD that have elevated mean expression compared to other neuronal genes. It is tempting to speculate that the high mean expression level of these genes identifies them as having crucial roles in maintaining normal brain function, their dysregulation causes ASD.

### Gene level: SFARI genes have smaller differences in level of expression between ASD and control patients than other neuronal genes

This section studies the relation between the SFARI genes and differential expression analysis between ASD and control groups by comparing the percentage of differentially expressed genes in each group as well as the magnitude of the log fold-change of the genes.

**Figure 3:**
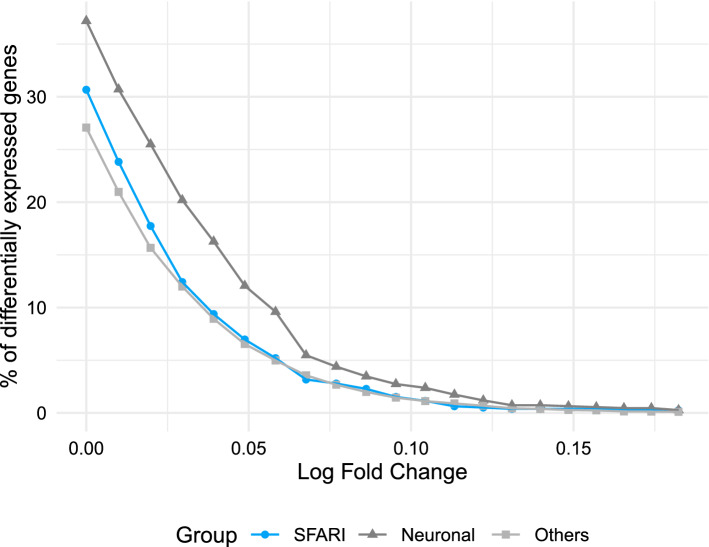
SFARI genes have a lower percentage of differentially expressed genes than neuronal genes and a similar percentage to the rest of the genes. Percentage of differentially expressed genes for different log fold-change thresholds grouping genes by SFARI, other neuronal genes, and the rest of the genes in the dataset. DESeq2 v1.24.0 https://bioconductor.org/packages/release/bioc/html/DESeq2.html.

We find that SFARI genes have a consistently lower percentage of differentially expressed genes when compared to the neuronal group, and very similar values to the rest of the genes, regardless of the log fold-change threshold (Fig. 3).

**Figure 4:**
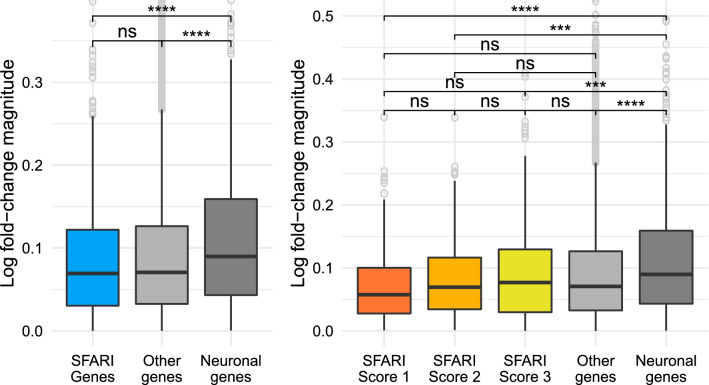
SFARI genes have lower log fold-change magnitudes than neuronal genes and similar magnitudes to non-neuronal genes. Comparison of the log-fold change magnitude between the SFARI genes, genes with neuronal annotations and with the rest of the genes in the dataset. As before, the asterisks at the top indicate the magnitude of the corrected p value from pairwise Welch t-test comparisons to study if the differences between groups is statistically significant. (**A**) SFARI genes. (**B**) SFARI Scores. Outlier genes are represented individually as open circles. The t-tests use all the points in each group, including outliers. DESeq2 v1.24.0 https://bioconductor.org/packages/release/bioc/html/DESeq2.html.

Comparing the log fold-change magnitude of genes in each category we find that the SFARI genes have statistically significantly lower values than genes with a neuronal function with a corrected p value lower than $10^{-4}$, and comparable log fold-change magnitudes to the remaining genes in the dataset (genes that are neither SFARI nor neuronal) (Fig. 4A).

Separating the SFARI genes by SFARI Scores we find that the higher the SFARI Score, the lower the log fold-change magnitude (Fig. 4B), with SFARI Score 1 having the lowest values of all groups, including the rest of the genes that are neither SFARI nor neuronal, followed by SFARI Score 2 and SFARI Score 3 genes having the highest. Differences in log-fold change between SFARI scores and between non-SFARI non-neuronal genes and each SFARI Score are visible but not statistically significant. However, differences between neuronal genes and all other gene groups are statistically significant with a corrected p value lower than $10^{-3}$.

### Module level: SFARI genes are not enriched in modules from gene co-expression networks that are strongly correlated with ASD diagnosis

In this section, the relation between SFARI genes and the modules obtained with WGCNA’s gene co-expression network is analysed, comparing each module’s association to the diagnosis status of the samples to their enrichment in SFARI genes to determine if there as a relation between them.

**Figure 5:**
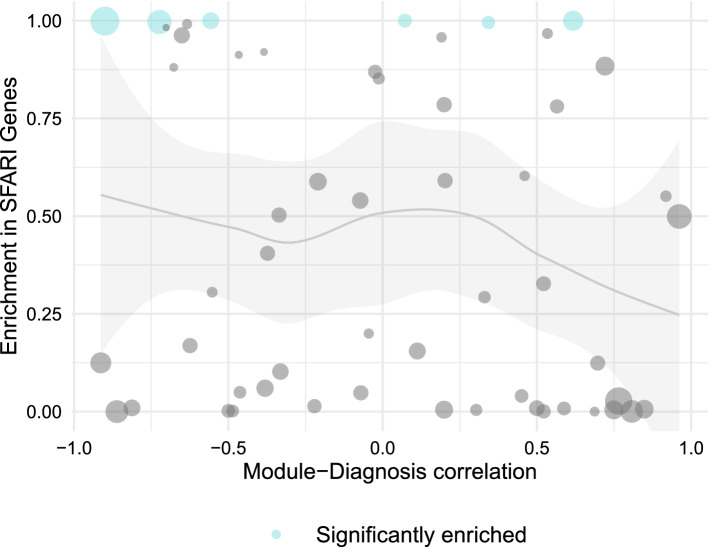
SFARI gene enrichment in modules does not correlate with ASD diagnosis status. Scatter plot of WGCNA modules comparing the strength of the correlation of the modules to the diagnosis of the samples and enrichment in SFARI genes. Each point represents a module; its position on the plane defined by the two metrics mentioned above, its size corresponds to the number of genes in the module, and its colour indicates if the enrichment in SFARI Scores is statistically significant using a corrected p value of 0.05. The grey line corresponds to the trend line illustrating the relation between the two variables we are studying, with the shaded area around the line displaying its 95% confidence interval. This means that with a 95% confidence the true trend connecting the two variables lies within this shaded area. WGCNA v1.69 https://cran.r-project.org/web/packages/WGCNA/index.html.

The network consist of 55 gene co-expression modules, with only 138 genes (0.9%) unassigned to any module. Measuring the association of a module to diagnosis using the module-diagnosis correlation and the enrichment in SFARI genes using Over Representation Analysis, we find that the distribution of modules that were found to be significantly enriched in SFARI genes is relatively uniform across different levels of module-diagnosis correlation; furthermore, enrichment in SFARI genes of all the modules is largely constant (Fig. 5). These findings suggest there is not a strong relationship between these two features.

**Figure 6:**
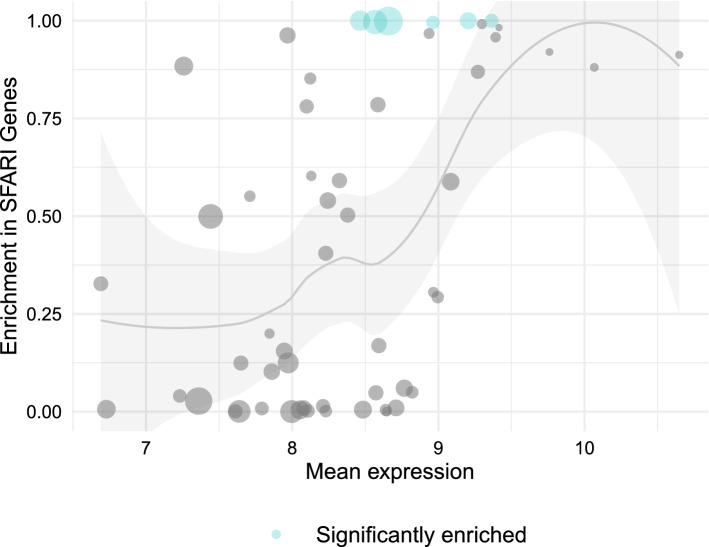
SFARI gene enrichment in modules is related to the mean level of expression of the genes in the module. Scatter plot of WGCNA modules comparing the mean level of expression of the genes contained in each module and the enrichment in SFARI genes. The details of the plot are the same as in Fig. 5. WGCNA v1.69 https://cran.r-project.org/web/packages/WGCNA/index.html.

Performing a similar analysis by substituting the module-diagnosis correlation of each module for the mean level of expression of the genes it contains, we get a much clearer pattern: as Fig. 6 shows, modules with higher levels of expression have a higher enrichment in SFARI genes, and none of the modules where the enrichment in SFARI genes were found to be statistically significant have a low mean level of expression. These results are consistent with the findings presented in the first section, and show that the positive relationship between level of expression and SFARI genes persist in WGCNA’s co-expression modules.

### Systems level: whole co-expression network integration facilitates discovery of novel candidate SFARI genes

This section studies the relation between SFARI genes and topological information extracted from the whole co-expression network to determine if there is a relation between the global structure of the network and the SFARI genes.

The previous sections show that local gene expression information is not robust enough to model ASD-related patterns from SFARI genes, but the fact that in the *Gene level* section these two data sources actively contradict each other, but in the *Module level* section they do not anymore, appearing to be independent, could indicate that integrating more information from the co-expression network had a positive effect. Based on this, we hypothesise that models built using more information from the whole co-expression network can represent more intricate shared patterns between genes and capture information that would remain hidden when studying genes at a more local level, allowing for the information coming from SFARI genes and from transcriptomic data to complement each other in a deeper way.

If this hypothesis is true, then we would expect SFARI genes to have a positive relation with the structure underlying the whole co-expression network, including its ASD-specific dysregulation patterns. To test this, we build a gene classifier using information extracted from the whole co-expression network including; the correlation between a gene’s expression pattern and diagnosis, and each module’s eigen-gene, and between a gene’s assigned network module and diagnosis, as descriptive variables, as well as a binary objective variable indicating whether the gene is a SFARI list gene. We obtain a probability for each non-SFARI gene that can be interpreted as how similar the gene is to the SFARI genes in the co-expression network. We can quantify the reliability of the model using the classifier’s performance metrics as well as analysing whether biological evidence exists in the literature to support the relevance to ASD of the genes with the highest probabilities.

The classification task was performed using a Ridge regression and the performance metrics selected were the Area Under the ROC Curve (AUC), the Maximum Lift Point (MLP) and the Balanced Accuracy which are described in “Methods” section. Table 1 shows the performance of this first classifier, referred to as the “original” model, and it can be seen that it has higher values for all three performance metrics than the “shuffled labels” model, which was used as a baseline against which we could compare our models. For this last model, the classifier used was the same Ridge regression, but the SFARI labels in the classification dataset are shuffled at random.

**Table 1:** Performance metrics of the two classification models used as well as a third model using a shuffling of the SFARI labels in the data.

Model	AUC	MLP	Balanced accuracy
Original	**0.69** $\mathbf {\pm 4 \times 10^{-4}}$	**20.43 ± 0**	**0.64 ± 0.0018**
Unbiased	0.58 ± 0.03	13.61 ± 6.84	0.56 ± 0.01
Shuffled labels	0.50 ± 0.02	2.83 ± 3.4	0.50 ± 0.01

The highest value for each performance metric is represented in bold.

The performance metrics of the “shuffled labels” model show that both the AUC and Balanced accuracy are 0.50 with small standard deviations, which means the model is not able to differentiate between classes at all, and although it has a MLP above 1, it has a very large standard deviation (larger than itself), so it means that this model may sometimes by chance have a large proportion of SFARI Genes in the top scoring genes but it is not reliable. All these metrics together indicate that the model, as expected, is not able to identify SFARI genes.

**Figure 7:**
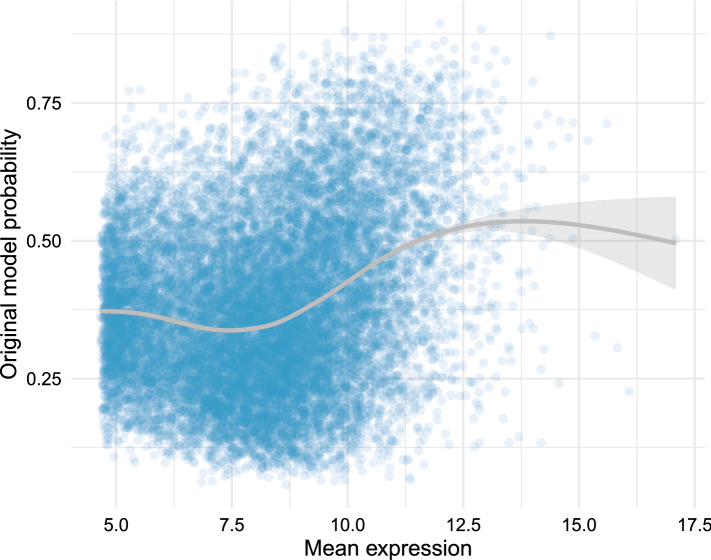
There is a positive relation between the level of expression of the genes and the probabilities assigned by the classification model. The x-axis corresponds to the mean level of expression of the genes and the y-axis to the probability assigned by the model indicating how likely they are to be SFARI genes. The grey line corresponds to the trend line illustrating the relation between these two features, with the shaded area around the line displaying its 95% confidence interval.

The original model performs well, as we can see in Table 1, but when we compare the mean expression of the genes against the probability assigned to them by this model, we find that there is a positive association between these two characteristics, with the genes with medium to high mean level of expression trending towards the upper-right part of Fig. 7. This suggests that the classifier is using the level of expression of a gene, or some confounder of it, as a factor when calculating its similarity to the SFARI genes, which was expected, since this relation had already been noticed both at gene- and module-level.

**Figure 8:**
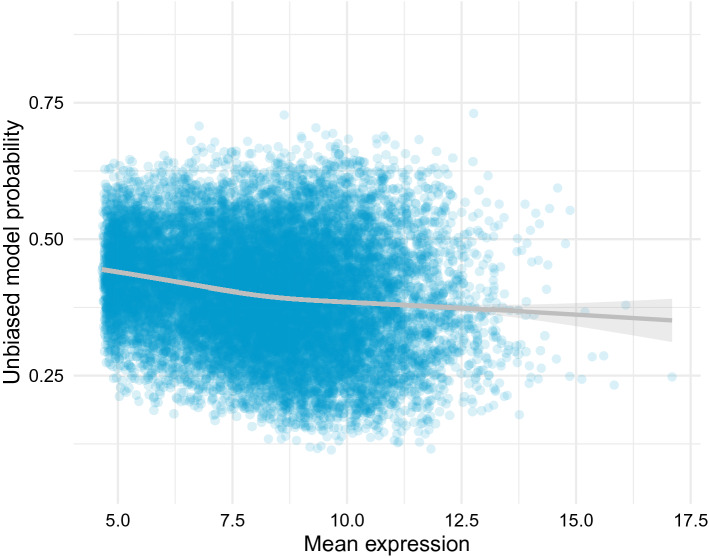
The bias correction algorithm removes the relation between the level of expression of the genes and the probabilities assigned by the classification model. Caret v6.0-86 https://cran.r-project.org/web/packages/caret/index.html Glmnet v3.0.2 https://cran.r-project.org/web/packages/glmnet/index.html.

As mentioned before, there is no biological evidence supporting the relation found between a gene’s level of expression and its role in ASD, so it is better to remove it from the model to be certain that the patterns it is detecting are genuinely biological. A bias correcting technique was used to correct this relation, after which the strongest patterns connecting the mean level of expression and the probability of the model are removed (Fig. 8), and only a small negative trend remains. This new version of the algorithm, which we call the “unbiased” model, has a worse performance than the original model, as seen in Table 1, because it is no longer using the mean expression of the genes to identify the SFARI genes, which was a strong indicator, but is still performing better than the “shuffled labels” model.

Table 2 shows the non-SFARI genes that were assigned the highest probabilities by this final unbiased model. All of these genes have been found to have some connection with ASD, and gene CORO1A has subsequently been included in the SFARI-gene list with a score of 1. This suggests the model is indeed able to identify genes with similar behaviour to SFARI genes and that the results also have biological relevance to ASD.

These results show that we can successfully identify novel candidate genes by combining a *systems-level* network approach to differential gene expression modelling with categorical labelling of disease genes, even when removing the signal related to the level of expression of the genes.

**Table 2:** Top 10 non-SFARI genes with the highest probabilities assigned by the unbiased model.

	Gene	Probability	Literature review
1	SNX25	0.73	CNV associated both to ASD and ADHD^44^
2	CLMP	0.71	QTN associated to play skills in twins with ASD^45^
3	EGR1	0.70	Role in the aberrant regulation of synaptic maturation in ASD^46^
4	HECTD2	0.69	Phylogenetically similar to UBE3A (SFARI Gene Score 1)^47^
5	PLXNC1	0.69	Part of the Axonal Guidance signaling pathway, one of the canonical pathways significantly associated with dysregulated genes with LINE-1 insertion^48^
6	AHI1	0.69	Mutations associated to ASD^49^
7	CORO1A	0.69	Now a SFARI Gene with Score 1 in the latest version of the dataset^5^
8	ARC	0.68	Target protein of gene UBE3A (SFARI Gene Score 1)^50^
9	ARPP21	0.68	Gene associated to candidate intergenic risk loci in ASD^51^
10	ARHGAP20	0.68	Differential expression related to ASD^52^

### Comparison with other scoring systems and disorders

Given the strong pattern related to the mean level of expression of the genes found in the SFARI genes dataset, this last section studies how pervasive this pattern may be, studying if it is also present in other lists of candidate ASD genes, or in genes believed to be involved in other neurodevelopmental disorders.

#### Other ASD scoring systems

Three ASD scoring systems were selected to compare against SFARI based on their popularity: the Krishnan probability score, which uses a gene co-expression network and a list of ASD genes (including the SFARI genes) to train a classifier; the Sanders TADA score, which uses whole-exome sequencing to incorporate information from de novo mutations, inherited variants present, and variants identified within cases and controls to create a gene-based likelihood model; and the DisGeNET score, which integrates information from various repositories (also including the SFARI genes). All of these scores are continuous instead of categorical like SFARI, so we use the Pearson correlation to make pairwise comparisons between these scores and the Polyserial correlation to compare them to the SFARI genes.

**Figure 9:**
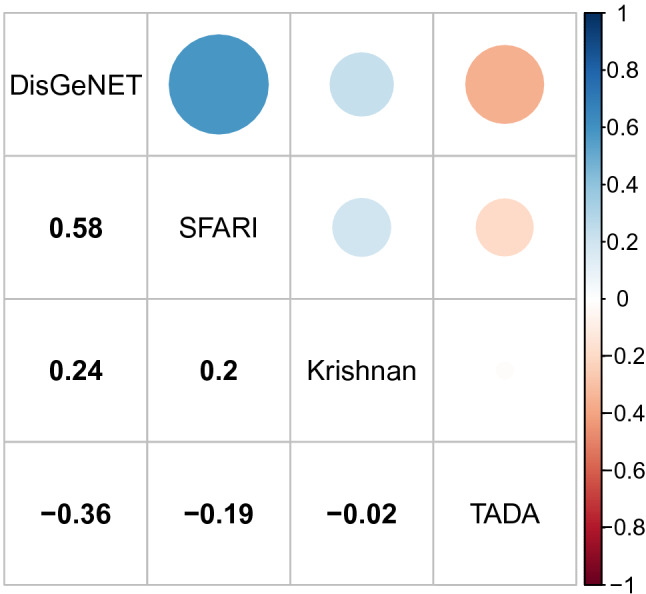
Pairwise correlation between the different ASD scoring systems. The size and colour of the circles correspond to the magnitude and sign of the correlation, respectively.

As Fig. 9 shows, the SFARI, DisGeNET and Krishnan scores have a strong correlation, while Sanders TADA score has either a neutral or a negative correlation with all the others. All of the correlations have a corrected p value lower than 0.05, the highest being Krishnan vs. TADA with 0.04.

Table 3 shows the correlation found between each of the scoring systems and the mean level of expression of the genes. Parallel to the results found above, The SFARI, Krishnan and DisGeNET scores have positive correlations, while Sanders TADA score appears to be independent.

The correlations between the SFARI, Krishnan and DisGeNET scoring systems as well as their statistically significant correlations with level of expression can be explained by the connections that exist between the SFARI-gene list and these other scoring systems, what is surprising is the strength and significance of these relations, which suggest that SFARI genes play a much more central role in the characterisation of these other scoring systems than expected.

**Table 3:** Correlation between different ASD scoring systems and the mean level of expression of the genes.

	SFARI	Krishnan	DisGeNET	TADA
Correlation	0.35	0.35	0.19	$-$ 0.01
p value	$4 \times 10^{-17}$	0	0.003	0.097

#### Relation between mean expression and other neuronal disorders

The gene scores for other neuronal disorders were obtained from DisGeNET. The disorders selected were Schizophrenia (Scz), Bipolar Disorder (BD), Intellectual Disability (ID), Depressive Disorder (DD) and Chronic Alcohol Intoxication (CAI).

A big proportion of the genes associated to all of these disorders belong to the SFARI genes, as Table 4 shows, the highest being Intellectual Disability with 24% and the lowest Schizophrenia, with 18%.

**Table 4:** Number of genes associated to different neuronal disorders according to DisGeNET and percentage of genes that belong to the SFARI genes list.

	ASD	Scz	BD	ID	DD	CAI
Total number of genes	231	765	415	425	254	228
% of SFARI genes	61.9%	18.0%	22.2%	24.2%	21.7%	18.9%

**Figure 10:**
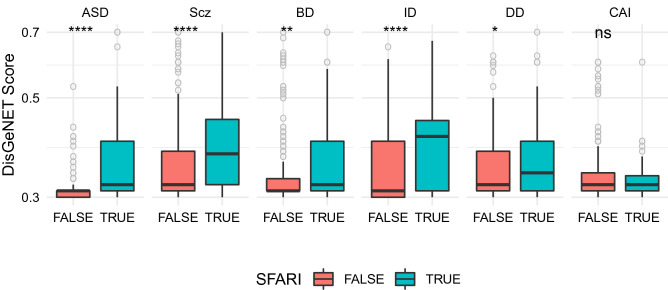
SFARI genes have higher DisGeNET Scores than the rest of the genes involved in different disorders. Box plots of gene scores from different disorders comparing SFARI genes with the rest of the genes. The asterisks at the top indicate how statistically significant is the difference between the two groups of genes.

Studying the scores associated to each of the disorders, Fig. 10 shows that SFARI genes are not only over-represented in all disorders, but they also have higher scores than the rest of the genes associated to each disorder. This difference is statistically significant for all disorders except for Chronic Alcohol Intoxication.

Finally, calculating the correlation between the different scores and the mean expression of the genes, Table 5 shows ASD is the disorder with the highest correlation, followed by Schizophrenia and Bipolar Disorder, all three of them with p values lower than 0.05, but this relation weakens significantly when we remove the SFARI genes, even for the genes related to ASD, as Table 6 shows, where Schizophrenia is the only disorder that still has a significant p value.

**Table 5:** Correlation between the scores associated to different disorders by DisGeNET and the mean level of expression of the genes.

	ASD	Scz	BD	ID	DD	CAI
Genes	231	765	415	425	254	228
Correlation	0.19	0.13	0.10	0.07	$-$ 0.07	$-$ 0.15
p value	0.003	0.0002	0.04	0.16	0.29	0.03

**Table 6:** Correlation between the scores associated to different disorders by DisGeNET, removing the SFARI genes, and the mean level of expression of the genes.

	ASD	Scz	BD	ID	DD	CAI
Non-SFARI genes	88	627	323	322	199	185
Correlation	0.02	0.09	0.07	$-$ 0.06	$-$ 0.08	$-$ 0.09
p value	0.86	0.02	0.24	0.31	0.24	0.20

Taken together these results demonstrate that the unexpected profile of the mean level of expression observed for genes in the SFARI-gene list permeates not only to other ASD scoring systems, but, because of the important role this group of genes play in other neurodevelopmental disorders, it also has an impact in other neurodevelopmental disorders, especially Schizophrenia and Bipolar Disorder.

## Discussion

SFARI genes have a lower percentage of differentially expressed genes as well as a lower log fold-change magnitude than non-SFARI genes with neuronal function, and when separating the SFARI genes by score, we find that the higher the SFARI Score, the lower the log fold-change magnitude of the genes. This decrease within SFARI Scores and between SFARI genes and neuronal genes is not explained by the observed bias by level of expression, since the shrunken log fold-change estimates were used for this analysis, which already account for this. A possible explanation could be that SFARI genes are more tightly regulated than other genes with neuronal function, with SFARI genes assigned a score of 1 having the tightest regulation of all.

Modules derived from our gene co-expression network showed no significant correlation between the module diagnosis status and module enrichment for SFARI genes. This suggests that even though SFARI genes do cluster together within modules, these modules are not especially disrupted by ASD. The bias by gene expression level in modules was unexpected, since the network was built using pairwise gene correlations, and the correlation metric is invariant to linear transformations, which could mean that there may be more factors involved in this, and the level of expression may only be a confounding factor for another underlying trait.

Contrary to the results observed at *gene-level* and *module-level*, we demonstrate that SFARI-gene status can be successfully used in combination with differential gene expression data when considered at the *systems-level*. This suggests that local information is not sufficient to describe the complex role SFARI genes play in gene-expression profiles and their dysregulation in ASD, but instead requires the whole network to model this intricate system.

The classifier used here was chosen for its explicit interpretability rather than predictive power per se, so it would be interesting in the future to determine whether different classification approaches are able to further improve on classification performance and to what extent this approach can generalise to other biological settings. Models could further be developed to embrace a semi-supervised learning approach because SFARI genes are confirmed disease genes, so it is valid to label them as positive, but the opposite is not true for non-SFARI genes since we do not know whether they are associated with ASD or not, so instead of labelling them as strictly negative, a better approach might be to leave them unlabelled, as the PU Learning methodology proposes^19^, and which has already been used for disease gene identification in protein–protein interaction networks with reported good performance^20^. We also consider that the selection of which features to extract and use from the co-expression network warrants further investigation since much of the information about the structure of the network is lost, so using a classifier directly on the network, as reported elsewhere^17^, could be productive in further optimising classification performance.

The relationship found between SFARI genes and the mean level of expression was significant and persisted throughout all of the levels of our analysis. Although we don’t know what could be causing this, a possible explanation for it, as well as for the bias within the SFARI Scores, could be a bias in the selection of the participants for genetic experiments; focusing mostly on people with moderate to severe ASD and overlooking people with milder cases, since^21^ found that the severity of ASD phenotype is directly related to the expression level of the genes, but since no information about the severity of the ASD of the participants is in the Spark Gene List^22^, on which the SFARI-gene selection and scoring criteria rely, we cannot assess this possibility.

Importantly, the bias found in the mean expression of SFARI genes is also present in other ASD gene scoring systems with the exception of the TADA-score. This observation could be an indirect effect of the incorporation of SFARI-gene related information into the generation of DisGeNET and Krishnan scoring systems, but it is not clear how this would result in such a strong effect based on how different are the methods by which the scores are calculated. Similarly, when we look at the DisGeNet scores of SFARI genes for other neuronal disorders, we find they have statistically significantly higher scores than the rest of the genes associated with each disorder. This raises the possibility that there may be significant shared molecular aetiology between these neurological diseases.

The relationship between SFARI genes and ASD-specific gene expression data is subtle and complicated, needing information derived from the whole gene co-expression network to be modelled accurately. We have shown that neither differential expression results nor co-expression modules with a high correlation to diagnosis status are significantly associated with SFARI genes. Rather, careful *systems-level* network analysis and the use of machine learning models to combine different sources of data in disease settings can prove to be highly effective at least for the novel candidate gene prediction approach addressed here. We also emphasise the importance of carefully studying the innate features of the gene expression data used in any given study as exemplified by the sizeable gene expression level feature found for SFARI genes and SFARI Scores which, to our knowledge, has been overlooked until now, and we propose a novel method to remove this pattern and study the effects this has. Understanding the intricate behaviour of SFARI genes is crucial, as their influence permeates to other ASD scoring systems, and even impacts data from other neurodevelopmental disorders. Further studies into the origins of this observed gene expression level bias and its origins will undoubtedly help us better understand ASD in the future.

## Methods

Pre-processing and analysis of transcriptomic data was performed using the DESeq2^23^ and WGCNA^24^ software packages, and the classification models using the caret^25^ and glmnet^26^ packages.

### Datasets

The version of the SFARI Gene dataset used corresponds to Q1 2020. It contains 1114 genes, of which 202 genes have a score of 1, 239 a score of 2 and 586 a score of 3. The 87 genes that were not assigned a score were not included in the analysis (Supplementary Information).

For the transcriptomic data, three RNA-seq datasets were studied, all consisting of human post-mortem brain tissue samples belonging to ASD individuals as well as a non-psychiatric control group. The main dataset was obtained from the GitHub repository from^27^. It contains 88 samples; 53 belonging to 24 ASD individuals and 35–17 controls, corresponding to the frontal, temporal, parietal and occipital cortical regions. After preprocessing, the final dataset contains 16132 genes and 80 samples. The first supporting dataset corresponds to^28^. It contains 104 samples; 47 belonging to 32 ASD individuals and 57–40 controls, extracted from the frontal and occipital lobes. The final version of this dataset contains 13,162 genes and 89 samples. And the second supporting dataset was obtained from^29^, the expression matrix was downloaded from^30^ and the metadata information from NCBI’s Gene Expression Omnibus^31^ with Series accession number GSE102741. It contains 52 samples, all corresponding to the dorsolateral prefrontal cortex; 13 of these belong to ASD individuals and 39 to controls. The final version of this dataset contains 15,392 genes and 49 samples.

Post-mortem samples may suffer from RNA degradation resulting from technical differences in sample collection and processing and as the length of time between death and sampling increases. These effects can be extremely heterogeneous and affect read quality and coverage. We used post mortem interval (2–43 h) and RNA integrity number (2.6–7.9) data to assess any impact on gene expression but observed no significant effects for the samples used in this study.

To broadly define genes that had neuronal functions we annotated genes as “Neuronal” using Gene Ontology annotations^32,33^ if their term name or description contained the substring “neuro”. All comparisons performed between SFARI genes and the rest of the genes within the gene expression data are performed separately, allowing us to compare SFARI genes to non-SFARI neuronal genes as well as to non-SFARI non-neuronal genes as required.

“Krishnan-scores” were obtained from genome-wide autism-gene predictions available from http://asd.princeton.edu as part of the supplementary material from^34^. “TADA-scores” were extracted from Table S3 in^35^, and “DisGeNET-scores” were retrieved using the disgenet2r R package^36^.

### Data preprocessing

Meta-data for genes were retrieved from NCBI^37^ using the bioMart package^38^. During filtering we retained known protein coding genes. Of these, genes with a high percentage of zeroes across all samples were removed. The threshold for this was determined as the minimum percentage of zeroes where the strongest heteroscedasticity patterns in the normalised dataset disappears, which was 75% for our main dataset. We next removed outlier samples by calculating the pairwise correlation between expression profiles, then aggregating these for each sample and calculating their distance to the rest of the samples as a group. Outlier samples were identified if this distance was larger than two standard deviations away from the mean.

For Differential Expression Analysis (DEA), first, the SVA package^39^ was used to calculate the surrogate variables associated with unknown sources of batch effects in the data, and then, the DESeq2 package was used to perform DEA, using Diagnosis as target and including the batch-related features as well as the surrogate variables obtained from SVA into the formula. The null hypothesis used for the analysis was a log fold-change threshold of 0. After this, the data was normalised using the *vst* function from the DESeq2 package. Finally, batch effects were corrected for using a linear transformation to remove the effects captured by the surrogate variables from the *SVA* and *ComBat* functions removing the batch effects captured by the original features of the samples.

**Figure 11:**
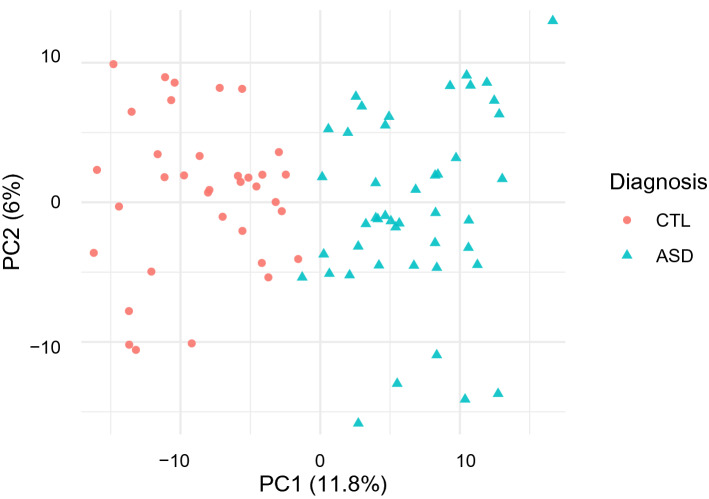
Diagnosis plays an important role in the characterisation of the samples. PCA plot of samples characterised by their expression patterns across all genes. This figure was created using the transpose of the matrix used for Fig. 1.

After preprocessing, the main feature that characterises our samples is their diagnosis status. This achieves perfect separation of the samples using only the first principal component (Fig. 11).

### WGCNA and enrichment analysis

The coexpression patterns of the genes were modelled using a network, which was built using the Weighted Gene Correlation Network Analysis (WGCNA) package: the expression matrix was transformed using the biweight midcorrelation metric, with the *signed hybrid* and *pickSoftThreshold* functions to obtain a scale-free topology, and subtracting the resulting topological overlap matrix from 1.0 to obtain the dissimilarity matrix. Clusters within this matrix were identified using hierarchical clustering with the *cutreeDynamic* algorithm. The strength of the relation between each of these modules and Diagnosis status was measured with the correlation of the module’s first principal component (called Eigengene) and the Diagnosis feature vector of the samples belonging to that module. Modules with a correlation magnitude higher than 0.9 were considered to have a strong correlation with the diagnosis status.

The enrichment in SFARI genes within a module was calculated using the Over Representation Analysis (ORA) provided by the clusterProfiler package^40^; the modules with a Bonferroni corrected p value lower than 0.05 were labelled as having a statistically significant enrichment in SFARI genes.

### Classification model

The dataset used to train the classification model consists of all the genes that were assigned to a module by WGCNA, characterised by a set of descriptive variables and a binary objective variable indicating if the gene is included in the SFARI-gene set or not, ignoring the SFARI Scores. The descriptive variables selected for the model are the correlation of a gene’s expression pattern to diagnosis status (called Gene Significance), including both the original correlation and its absolute value; the correlation of a gene’s assigned module to diagnosis status (called Module-Trait correlation); and the gene’s correlation to the eigen-gene of each of the modules in the network (called Module Membership). The resulting dataset consists of 15,994 observations, 58 descriptive variables, and one objective variable, which contains 789 positive and 15,211 negative values.

The genes are separated into training and testing sets, using 75% of the genes in the training set, where the imbalance between labels is corrected using the SMOTE over-sampling technique^41^, and reserving the remaining 25% of the genes for the test set.

Ridge regression^42^ was selected as the classification model because of the strong multicollinearity found in the descriptive variables in the dataset, using repeated cross validation to estimate the optimal value for the regularisation parameter of the model using 10-fold cross validation with 5 repeats. The model is trained 100 times using different partitions of the training and testing sets and the results from each of the runs are combined for the calculation of the final predictions and performance evaluation of the model. The performance metrics used are; (1) area under the ROC curve (AUC), which measures the ability of a classifier to distinguish between classes by comparing the true positive classification rate and the false positive classification rate at different probability thresholds. A value of 0.5 indicates that the model cannot distinguish between classes at all and a value of 1.0 reflects a perfect separation. (2) Maximum lift point (MLP) which measures the proportion of positive observations in the set of observations with the highest assigned probabilities against the proportion of positive samples in the entire dataset. A value of 1.0 indicates that the model does not assign the highest probabilities to the positive samples any more frequently than to the rest of the samples; increasing values greater than 1.0 reflect increasing model performance. (3) Balanced Accuracy, is a commonly used substitute for the regular Accuracy metric when classes are imbalanced, and is the average of the proportion of correctly classified positive observations. A value of 0.5 indicates that the model is no better than classifying observations at random and a value of 1.0 corresponds to a perfect classification.

As a modification to this regression model, the weighting technique proposed by^43^ was used to correct the bias found related to the mean level of expression of the genes. This technique focuses on the samples that are classified as a specific category (in our case as SFARI genes), measures the bias in each of them and, based on this, assigns a specific weight to all of the samples for the classifier to incorporate when re-training the model, giving larger weights to samples that do not have the bias and smaller weights to samples that reinforce it. This process of bias measurement, weight adjustment and re-training of the classification model is repeated iteratively until the bias becomes negligible.

For the implementation of this technique, Demographic Parity was used to measure the bias, which considers a classifier to be fair when it makes positive predictions in each segment of the population at the same rate as in all the population, and since this technique was designed for biases associated to categorical variables (such as gender or ethnicity) and our bias is a continuous one, some alterations had to be made to the constraint that measures the bias so that it could reflect its magnitude in a continuous instead of a binary way. The constraint selected for this was:

1 $$\begin{aligned} c(x,1)=\frac{\text {MeanExpression}(x) - mean(\text {MeanExpression}(G))}{sd(\text {MeanExpression}(G))} \end{aligned}$$

where *x* corresponds to each of the genes that are labelled as SFARI genes by the model in the previous iteration and *G* to all of the genes in the dataset.

**Figure 12:**
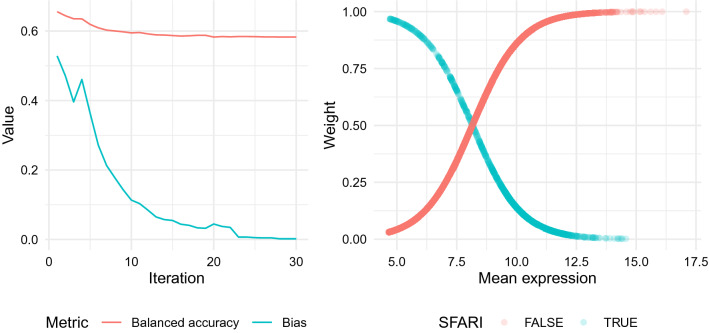
The bias correction algorithm removes the bias in the model by adjusting the weights of the samples in an optimal way. (**A**) Balanced accuracy and bias through each of the iterations of the bias correction algorithm. (**B**) Weights assigned to each gene by the final iteration of the bias correction algorithm based on their mean expression and label. Caret v6.0-86 https://cran.r-project.org/web/packages/caret/index.html Glmnet v3.0.2 https://cran.r-project.org/web/packages/glmnet/index.html.

Figure 12A shows how the bias correction algorithm removes the bias completely while barely affecting the performance of the model, and Fig. 12B shows how the model assigns high weights to samples that contradict the bias, such as non-SFARI genes with high levels of expression and SFARI genes with low levels of expression, and lower weights to the samples that reinforce it.

The top candidate gene list comprises those genes with the highest probabilities assigned by the final model and represent genes that share features in common with existing SFARI-genes. To allow calculation of the standard deviation of the performance metrics, the whole model, including the repetitions for different training-testing partitions, is repeated 100 times using different random seeds.

## Supplementary Information

Supplementary Information.

## Data Availability

Data are available from the Edinburgh DataShare repository https://doi.org/10.7488/ds/2980 and the source code from GitHub (https://doi.org/10.5281/zenodo.4463693).
