# Supplementary material for: SFARI genes and where to find them; modelling Autism Spectrum Disorder specific gene expression dysregulation with RNA-seq data
Source: Sci Rep. 2022 Jun 16;12:10158. doi: 10.1038/s41598-022-14077-1 (PMC9203566; doi:10.1038/s41598-022-14077-1)
Supplement: Supplementary file 1 — Supplementary Information. [file 41598_2022_14077_MOESM1_ESM.pdf]

Supplementary Figure Legends

Supplementary Figure S1. Mean level of expression plays a central role in gene characterisation
The mean level of expression is related to over 94% of the variance in all three datasets. A: Gupta's dataset. B: Wright's dataset.

Supplementary Figure S2. SFARI genes have higher levels of expression than other genes
All observed patterns persist except for Gupta's dataset, where SFARI Genes have lower level of expression than genes with neuronal annotations. A: SFARI Genes in Gupta's dataset. B: SFARI Scores in Gupta's dataset. C: SFARI Genes in Wright's dataset. D: SFARI Scores in Wright's dataset.

Supplementary Figure S3. SFARI Genes have a lower percentage of differentially expressed genes than neuronal genes and a similar percentage to the rest of the genes
This pattern can also be found in Gupta's dataset. This figure only contains information from Gupta's dataset because Wright's dataset does not have enough differentially expressed genes to create this plot.

Supplementary Figure S4. SFARI genes have lower log fold-change magnitudes than neuronal genes and similar magnitudes to non-neuronal genes
The previously observed patterns remain, except for SFARI genes in Wright's dataset, which tend to have lower log-fold change magnitudes than both neuronal genes and the rest of the genes, and the differences in log fold-change between SFARI scores in Gupta's dataset, which are not as clear as in the other two datasets. A: SFARI Genes in Gupta's dataset. B: SFARI Scores in Gupta's dataset. C: SFARI Genes in Wright's dataset. D: SFARI Scores in Wright's dataset.

Supplementary Figure S5. SFARI gene enrichment in modules does not correlate with ASD diagnosis status
There is no consistent pattern relating the module-diagnosis correlation and the enrichment in SFARI genes in all three datasets. A: Gupta's dataset. B: Wright's dataset.

Supplementary Figure S6. SFARI gene enrichment in modules is related to the mean level of expression of the genes in the module
There is a positive relation between mean expression and enrichment in SFARI genes by module in all three datasets. A: Gupta's dataset. B: Wright's dataset.

Supplementary Figure S7. There is a positive relation between the level of expression of the genes and the probabilities assigned by the classification model
Gupta's classification model does not have a bias as strong as the other two models related to the level of expression of the genes. A: Gupta's dataset. B: Wright's dataset.

Supplementary Figure S8. The bias correction algorithm removes the relation between the level of expression of the genes and the probabilities assigned by the classification model
The strongest bias by level of expression have been removed from all classification models. A: Gupta's dataset. B: Wright's dataset.

Supplementary Figure S9. Diagnosis plays an important role in the characterisation of the samples
Gandal's dataset has the cleanest separation between diagnosis groups, but a partial separation can be observed in all three datasets. A: Gupta dataset. B: Wright dataset.

Supplementary Figure S10. The bias correction algorithm removes the bias in the model by adjusting the weights of the samples in an optimal way
The bias correcting algorithm removes the bias from all classification models by assigning weights to SFARI genes inversely proportional to their level of expression and directly proportional to the rest of the genes. Consistent with Supplementary Figure 7, Gupta's dataset has the smallest bias of the three datasets. A: Balanced accuracy and bias through each of the iterations of the bias correction algorithm for Gupta's dataset. B: Weights assigned to each gene by the final iteration of the bias correction algorithm for Gupta's dataset. C: Balanced accuracy and bias through each of the iterations of the bias correction algorithm for Wright's dataset. D: Weights assigned to each gene by the final iteration of the bias correction algorithm for Wright's dataset.

Supplementary Tables

Dataset	Model	AUC	MLP	Balanced Accuracy
Gupta	Original	**0.69** $\pm\mathbf{8\times10^{-4}}$	10.55 ± 1.40	**0.64 ± 0.0029**
	Unbiased	0.67 ± 0.01	**16.72 ± 0**	0.62 ± 0.01
	Shuffled labels	0.50 ± 0.02	3.31 ± 3.68	0.50 ± 0.01
Wright	Original	**0.67** $\pm\mathbf{8\times10^{-4}}$	**11.66 ± 2.90**	**0.59 ± 0.0024**
	Unbiased	0.56 ± 0.02	4.30 ± 3.54	0.51 ± 0.01
	Shuffled labels	0.50 ± 0.01	3.02 ± 3.81	0.50 ± 0.007

Supplementary Table S1. Performance metrics of the two classification models used as well as a third model using a shuffling of the SFARI labels in the data in Gupta's and Wright's datasets. The performance of the unbiased classification model in Wright's dataset is only marginally better than the random model.

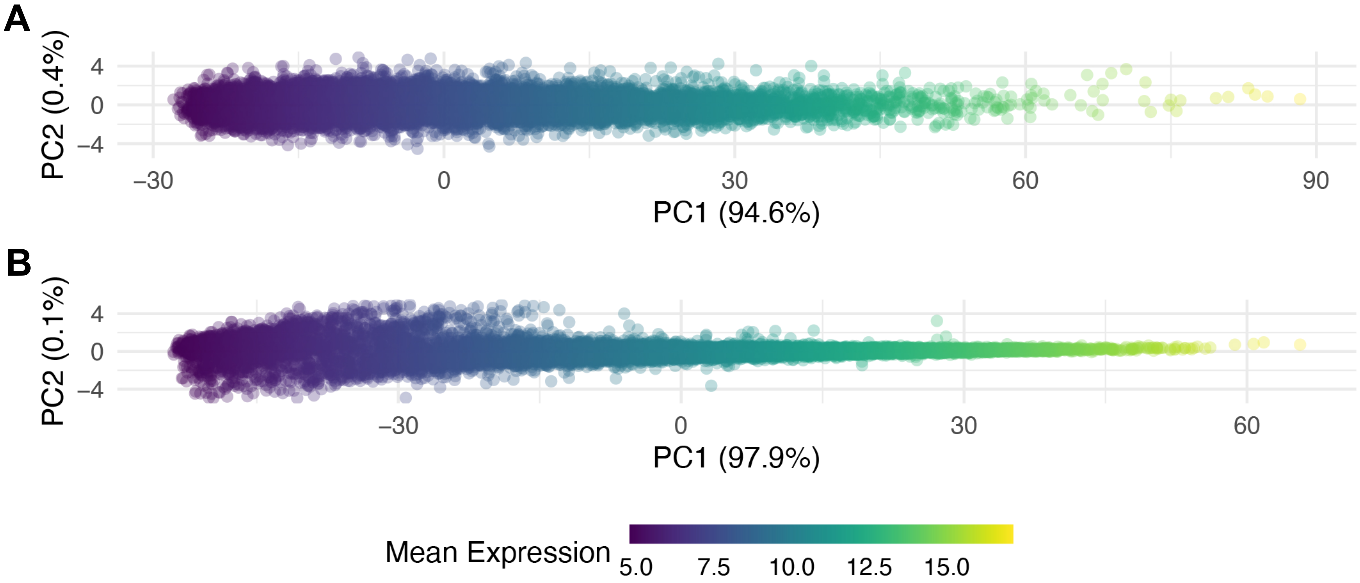
A
PC2 (0.4%)
4
0
−4
−30
0
30
60
90
PC1 (94.6%)
B
PC2 (0.1%)
4
0
−4
−30
0
30
60
PC1 (97.9%)
Mean Expression
5.0
7.5
10.0
12.5
15.0

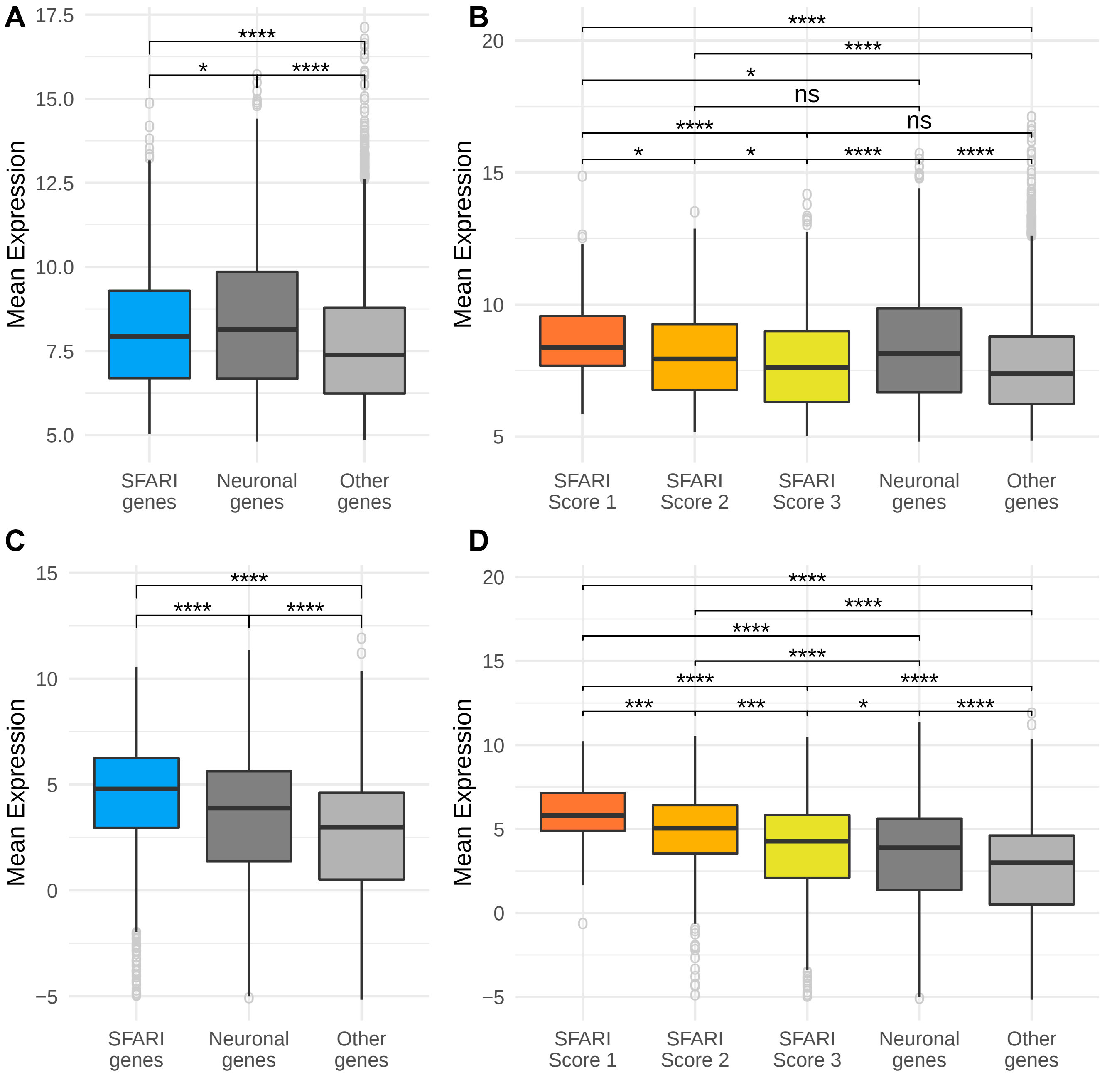

A
Mean Expression
17.5
15.0
12.5
10.0
7.5
5.0
****
*
****
SFARI genes
Neuronal genes
Other genes
B
Mean Expression
20
15
10
5
****
****
*
ns
****
ns
*
*
****
****
SFARI Score 1
SFARI Score 2
SFARI Score 3
Neuronal genes
Other genes
C
Mean Expression
15
10
5
0
−5
****
****
****
SFARI genes
Neuronal genes
Other genes
D
Mean Expression
20
15
10
5
0
−5
****
****
****
****
****
****
***
***
*
****
SFARI Score 1
SFARI Score 2
SFARI Score 3
Neuronal genes
Other genes

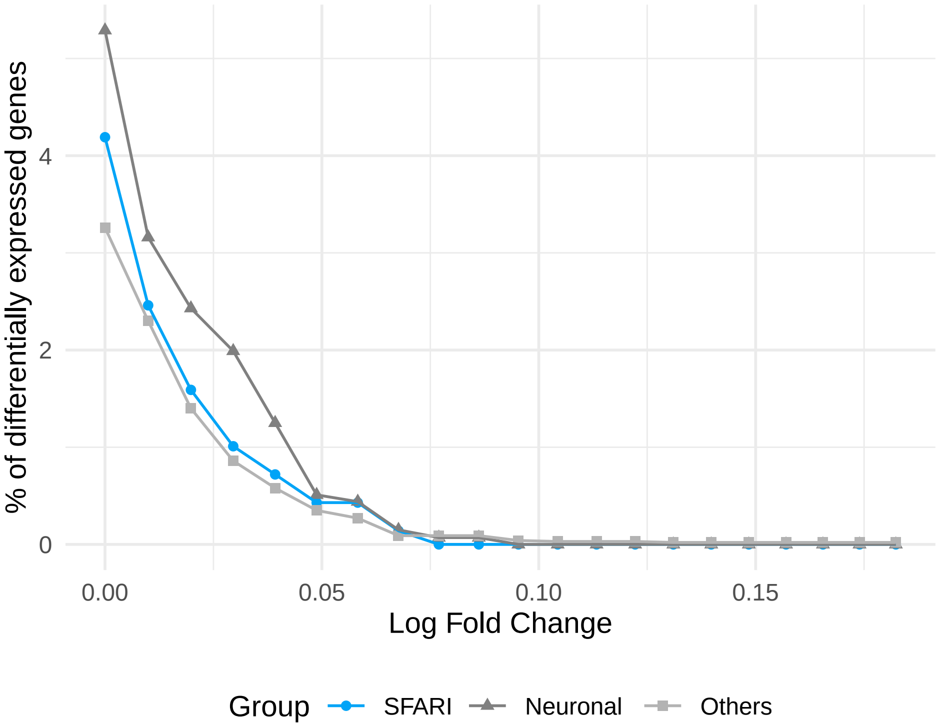

% of differentially expressed genes
0
2
4
0.00
0.05
0.10
0.15
Log Fold Change
Group
SFARI
Neuronal
Others

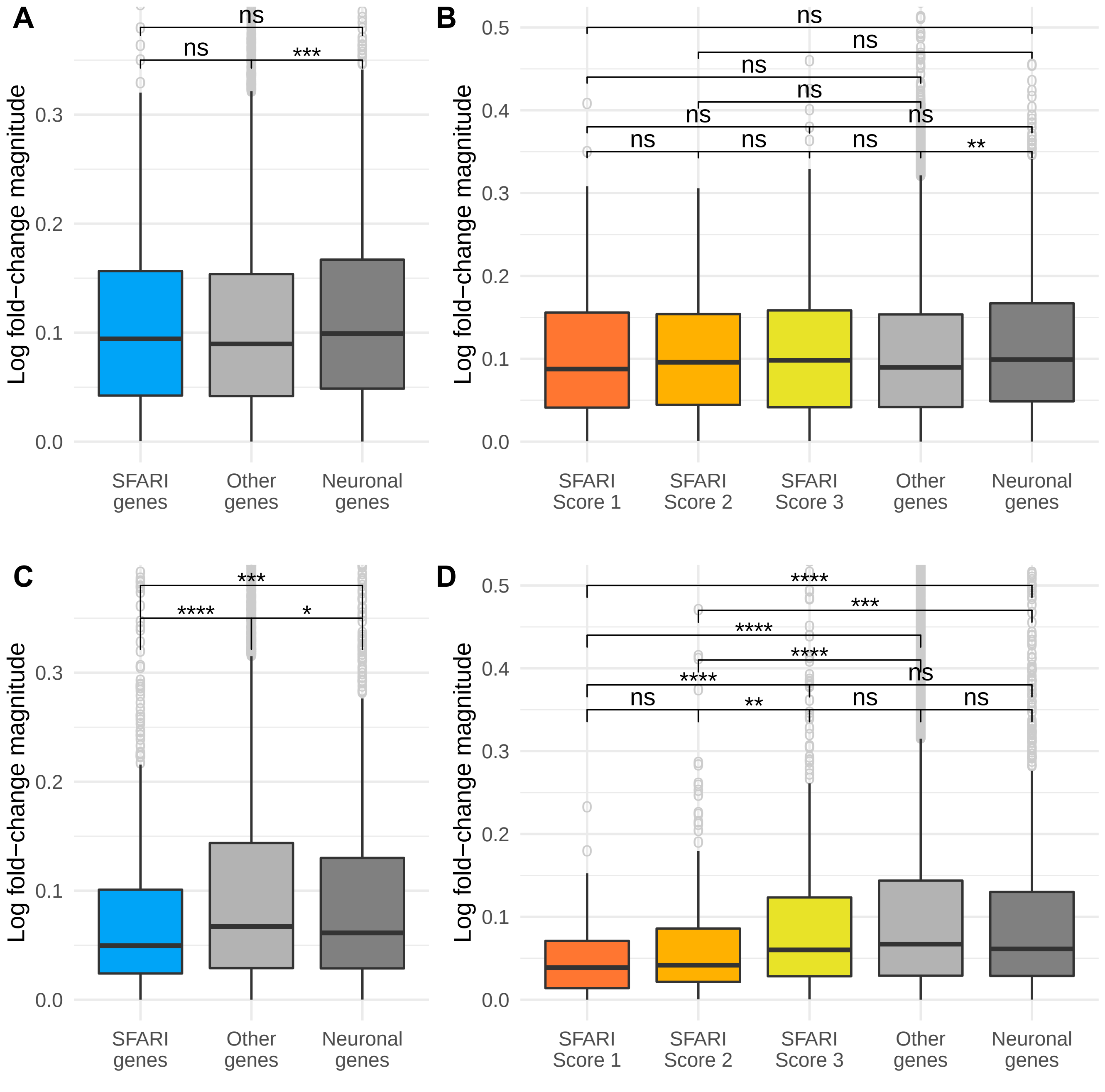

A
B
C
D
Log fold−change magnitude
0.0
0.1
0.2
0.3
0.4
0.5
ns
***
****
**
*
SFARI genes
Other genes
Neuronal genes
SFARI Score 1
SFARI Score 2
SFARI Score 3

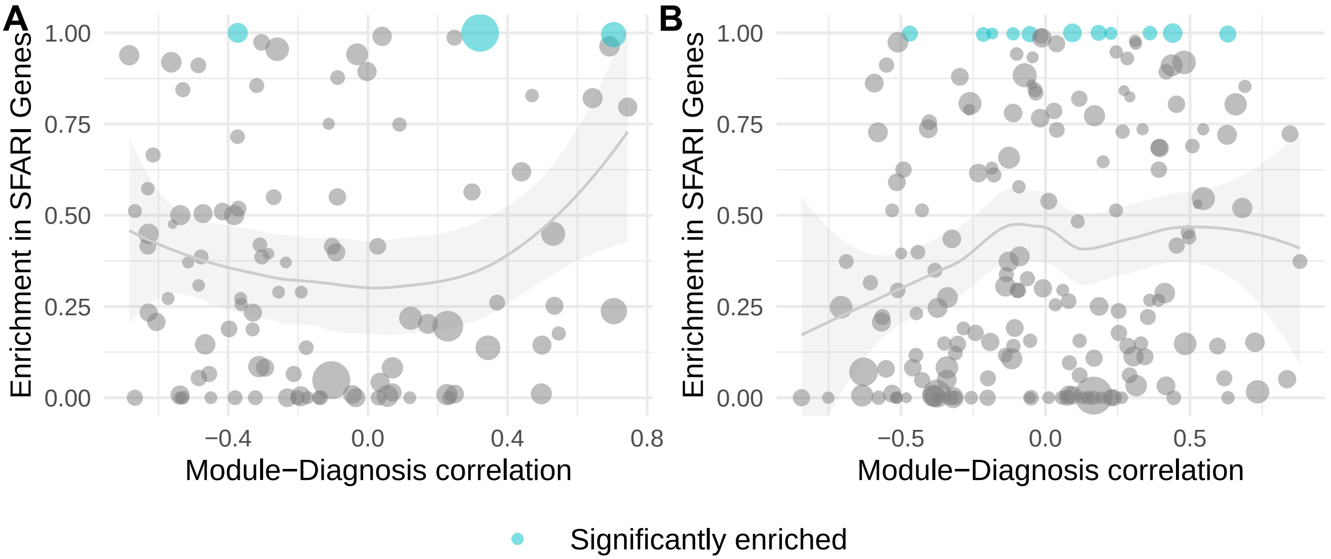
A
B
Enrichment in SFARI Genes
1.00
0.75
0.50
0.25
0.00
−0.4
0.0
0.4
0.8
−0.5
0.0
0.5
Module−Diagnosis correlation
Significantly enriched

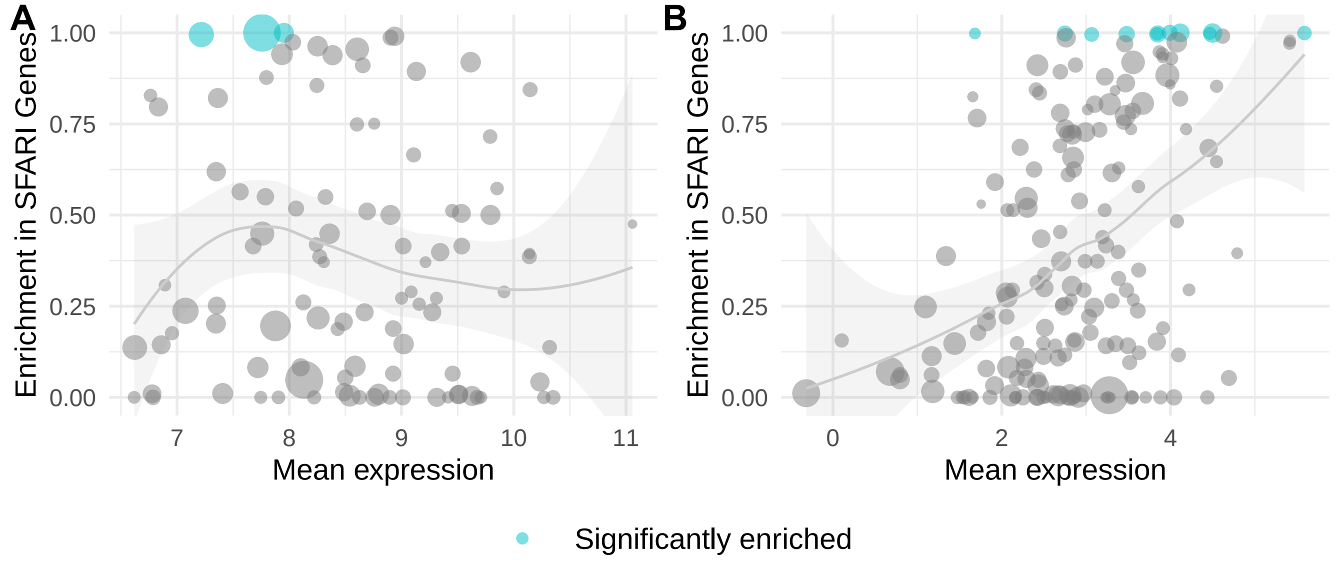
A
B
Enrichment in SFARI Genes
1.00
0.75
0.50
0.25
0.00
7
8
9
10
11
0
2
4
Mean expression
Significantly enriched

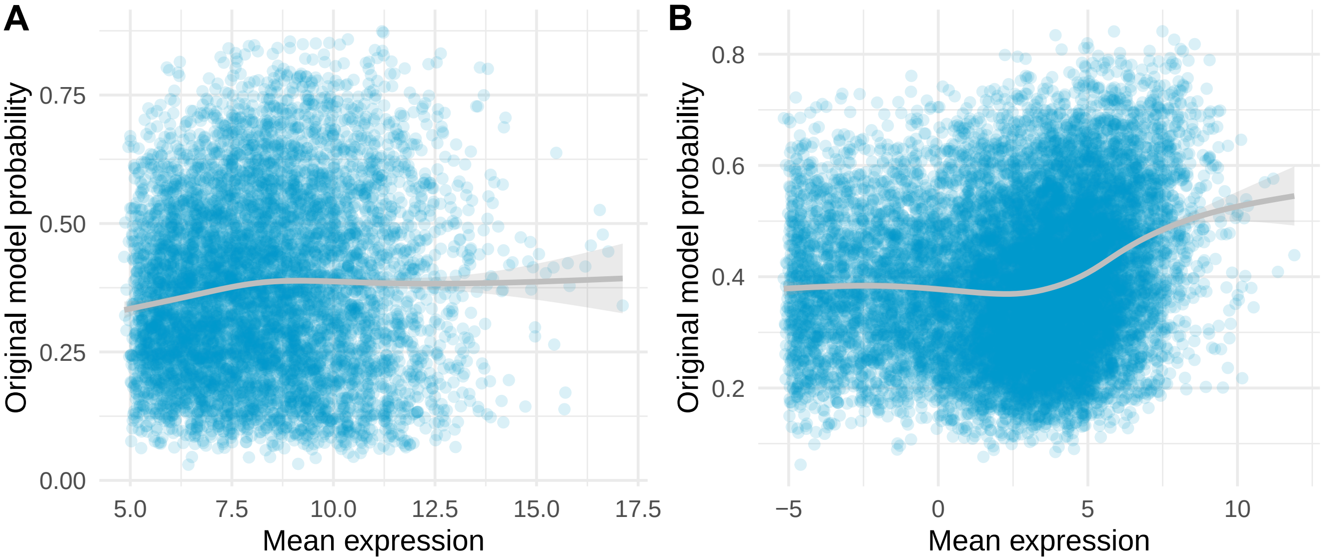

A
B
Original model probability
0.00
0.25
0.50
0.75
5.0
7.5
10.0
12.5
15.0
17.5
Mean expression
0.2
0.4
0.6
0.8
−5
0
5
10
Original model probability
Mean expression

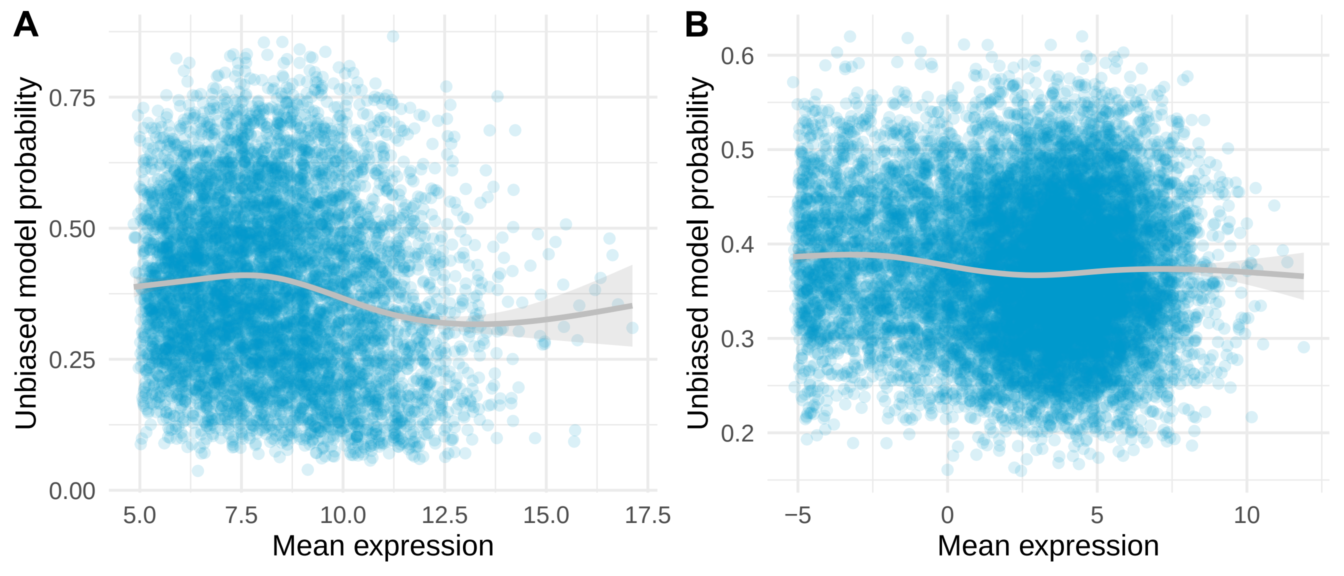
A
B
Unbiased model probability
0.00
0.25
0.50
0.75
5.0
7.5
10.0
12.5
15.0
17.5
Mean expression
0.2
0.3
0.4
0.5
0.6
−5
0
5
10

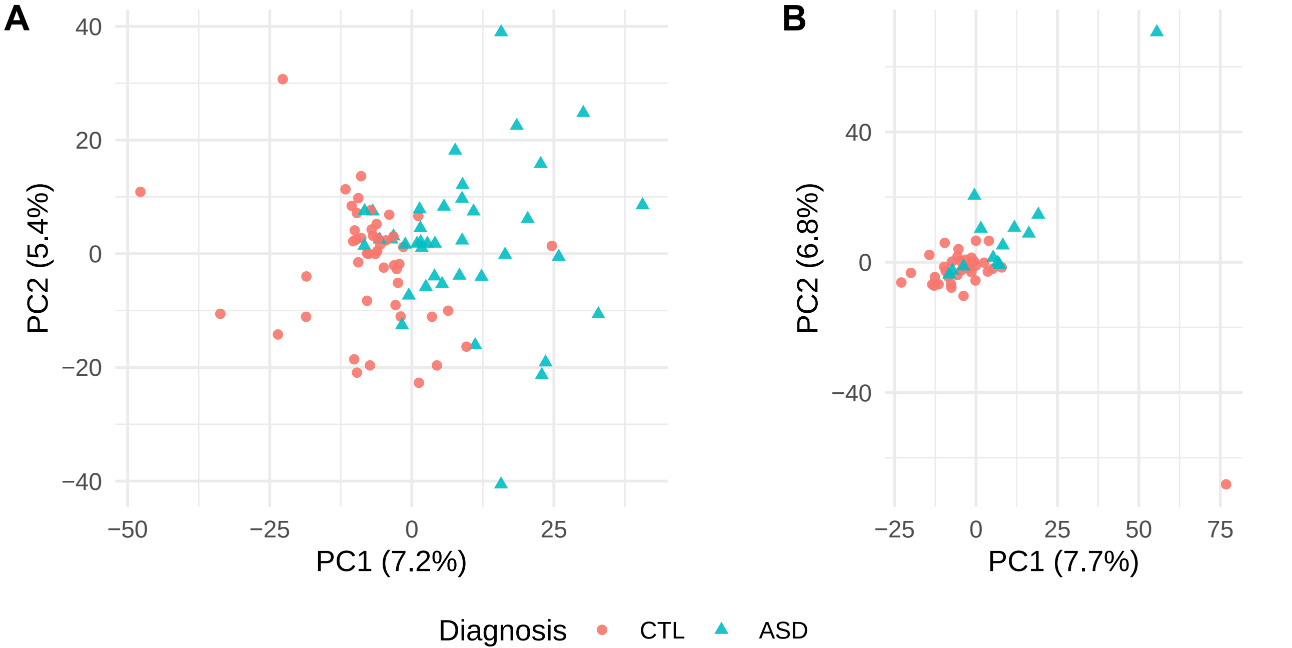
A
B
40
20
0
−20
−40
−50
−25
0
25
PC1 (7.2%)
PC2 (5.4%)
40
0
−40
−25
0
25
50
75
PC1 (7.7%)
PC2 (6.8%)
Diagnosis
CTL
ASD

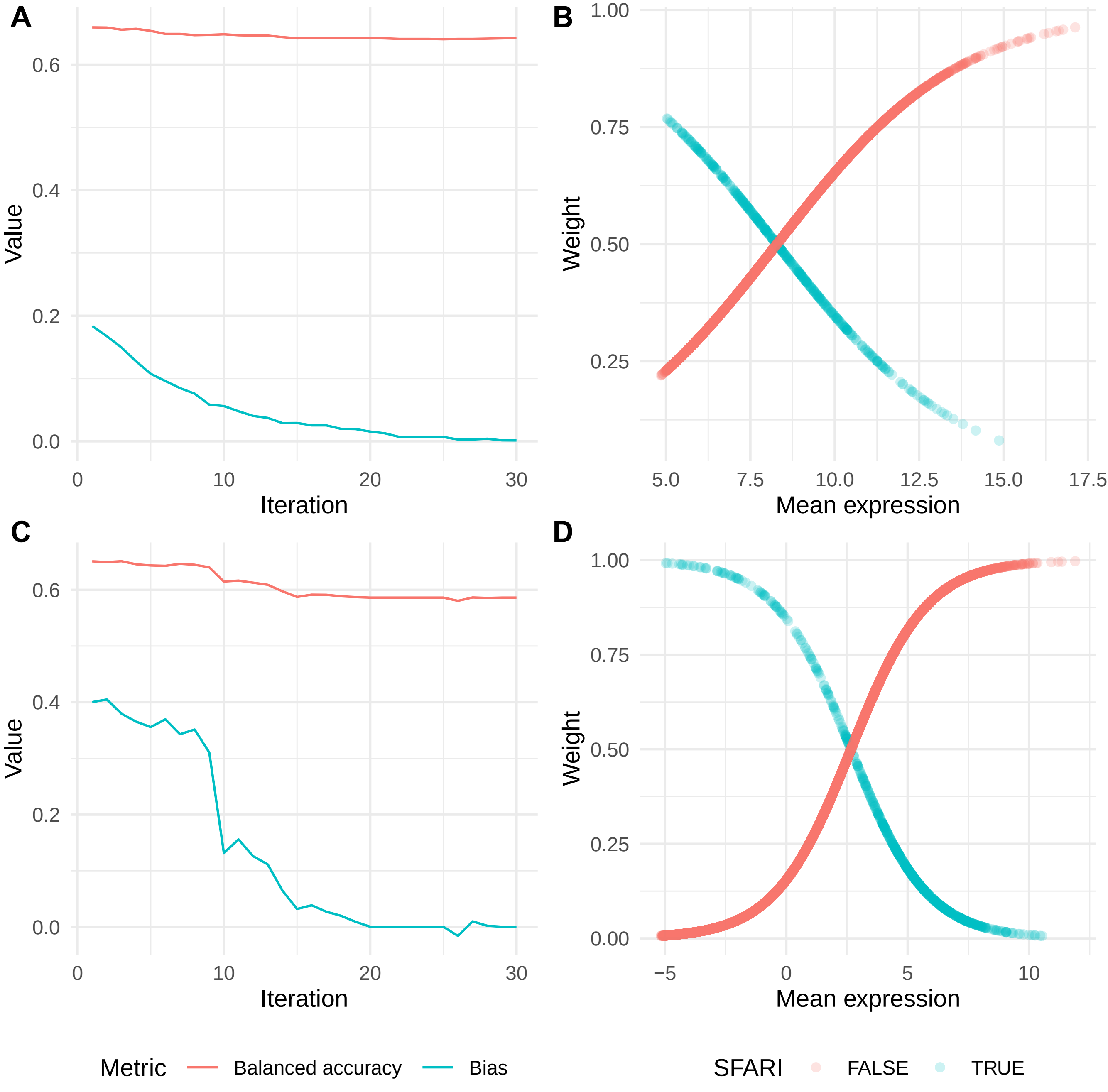

A
B
C
D
Value
Iteration
Weight
Mean expression
0.0
0.2
0.4
0.6
0
10
20
30
0.25
0.50
0.75
1.00
5.0
7.5
10.0
12.5
15.0
17.5
0.00
−5
5
Metric
Balanced accuracy
Bias
SFARI
FALSE
TRUE
